# Neoadjuvant tamoxifen synchronizes ERα binding and gene expression profiles related to outcome and proliferation

**DOI:** 10.18632/oncotarget.8983

**Published:** 2016-04-25

**Authors:** Tesa M. Severson, Ekaterina Nevedomskaya, Justine Peeters, Thomas Kuilman, Oscar Krijgsman, Annelot van Rossum, Marjolein Droog, Yongsoo Kim, Rutger Koornstra, Inès Beumer, Annuska M. Glas, Daniel Peeper, Jelle Wesseling, Iris M. Simon, Lodewyk Wessels, Sabine C. Linn, Wilbert Zwart

**Affiliations:** ^1^ Division of Molecular Pathology, Netherlands Cancer Institute, Amsterdam, CX, The Netherlands; ^2^ Division of Molecular Carcinogenesis, Netherlands Cancer Institute, Amsterdam, CX, The Netherlands; ^3^ Agendia NV, Amsterdam, XH, The Netherlands; ^4^ Division of Molecular Oncology, Netherlands Cancer Institute, Amsterdam, CX, The Netherlands; ^5^ Department of Medical Oncology, Radboud University Medical Center, Nijmegen, GA, The Netherlands; ^6^ Division of Medical Oncology, Netherlands Cancer Institute, Amsterdam, CX, The Netherlands; ^7^ Department of Pathology, University Medical Center Utrecht, CX, The Netherlands

**Keywords:** ChIP-seq, estrogen receptor, endocrine therapy, neoadjuvant therapy, gene expression analysis

## Abstract

Estrogen receptor alpha (ERα)-positive breast cancers are frequently treated with tamoxifen, but resistance is common. It remains elusive how tamoxifen resistance occurs and predictive biomarkers for treatment outcome are needed. Because most biomarker discovery studies are performed using pre-treatment surgical resections, the effects of tamoxifen therapy directly on the tumor cell *in vivo* remain unexamined. In this study, we assessed DNA copy number, gene expression profiles and ERα/chromatin binding landscapes on breast tumor specimens, both before and after neoadjuvant tamoxifen treatment. We observed neoadjuvant tamoxifen treatment synchronized ERα/chromatin interactions and downstream gene expression, indicating that hormonal therapy reduces inter-tumor molecular variability. ERα-synchronized sites are associated with dynamic FOXA1 action at these sites, which is under control of growth factor signaling. Genes associated with tamoxifen-synchronized sites are capable of differentiating patients for tamoxifen benefit. Due to the direct effects of therapeutics on ERα behavior and transcriptional output, our study highlights the added value of biomarker discovery studies after neoadjuvant drug exposure.

## INTRODUCTION

Breast cancer is the most common cancer among women in the world. In 2012 over 1.6 million women were diagnosed with breast cancer worldwide. In the same year, around 520,000 women died from breast cancer [1]. The inter-patient heterogeneous nature of breast cancer is evident and clinically relevant histological and molecular subtypes of breast cancer can be identified. [2 9]. The major subtype—estrogen receptor alpha (ERα)-positive luminal breast cancer—is clinically defined by nucleic protein levels of ERα [10]. ERα is a ligand-dependent transcription factor, activated by the natural hormone estradiol. Ligand-bound ERα can bind the DNA and recruit co-factors that form the foundations of the transcription complex, ultimately affecting target gene expression and driving tumor cell proliferation [11]. ERα-positive breast cancer has specific clinicopathological characteristics including a favorable prognosis compared with ERα negative disease, particularly in the first few years following diagnosis [12] and more often low tumor grade in comparison with other breast cancer subtypes [4]. Patients with ERα-positive breast cancer make up over 70% of the total breast cancer population worldwide [13].

Since ERα is the major driver in luminal breast cancers, endocrine therapies have been developed to limit its transcriptional potency, including tamoxifen and aromatase inhibitors [14]. Tamoxifen is aimed to competitively bind the ERα, which prevents co-factor recruitment and disrupts the transcriptional complex formation [15]. Through this mechanism, tamoxifen blocks ERα-responsive gene expression and inhibits ERα-positive tumor cell proliferation [16]. Aromatase inhibitors inhibit the production of estrogen and consequently inhibit tumor cell proliferation [17]. Although these endocrine therapies are successful agents, resistance to treatment is common. However, after developing a relapse despite tamoxifen treatment, 50% of patients still do respond to aromatase inhibitors [18]. Analogous to this, 50% of patients with acquired resistance to aromatase inhibitors still respond to tamoxifen [18]. This study and other reports [13] illustrate the heterogeneous response to first and second line endocrine therapy in ERα-positive breast cancer. Critically, these studies also exemplify that development of reliable predictive biomarkers for selective treatment efficacy is an urgent medical need.

From a clinical perspective, identification of patients most likely to benefit from specific endocrine treatments in the early ERα-positive breast cancer setting is paramount. Currently, biomarker discovery is guided by data (*e.g*. gene expression signatures) collected from the untreated primary tumor samples obtained during surgery. However, the prediction of longitudinal therapy response may be more reliable when data are available from samples after drug exposure. In the endocrine therapy setting, neoadjuvant studies have been used successfully to determine response [19, 20], indicating the potential of molecular data taken from post-treatment samples to gain clinical knowledge. Here, we present the first combined characterization of transcriptomic, epigenomic, genomic and clinical data analyzed from ERα-positive breast cancers both before and after 2-6 weeks of neoadjuvant tamoxifen treatment. By integrating these genomic data-streams from different time points, we aimed to examine drug-induced differences at the genetic, epigenetic and transcriptional level with the goal to identify novel predictive biomarkers for tamoxifen-response.

## RESULTS

### AFTER study and patient characteristics

**Figure 1:**
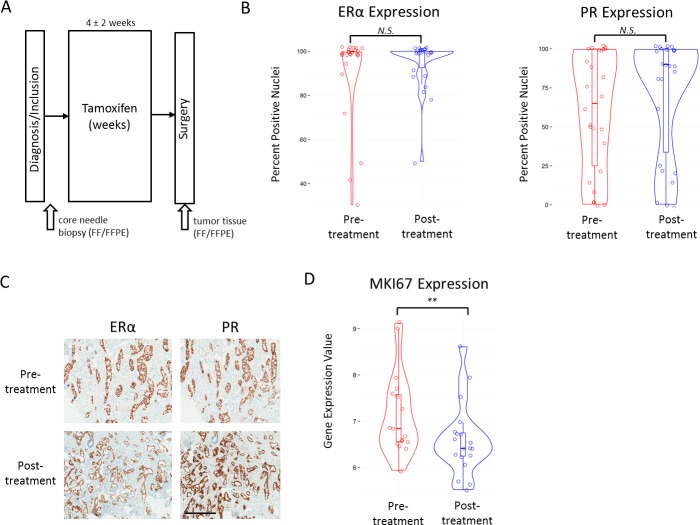
Study collection and clinical marker values **A.**, Schematic representation of the AFTER study patient and material collection. **B.**, Violin plots with boxplot overlay of IHC scores (Y-axis) for ERα (left panel) and PR (right panel) in pre- and post-treatment samples. N.S. indicates a *p*-value that is not significant at 0.05 level. **C.**, Example of IHC staining of ERα and PR in a pre- and post-treatment sample. Black bar indicates 400μm. **D.**, Violin plots with boxplot overlay of MKI67 gene expression values in pre- and post-treatment samples. ** indicates *P* < 0.01.

Forty-eight ERα-positive patients were enrolled at two hospitals in the Netherlands and sample material was taken from 28 tamoxifen-treated patients for further analysis (Figure 1A, Supplemental Figure 1A, 1B). Patient characteristics and collection data can be found in Table I. Patient treatment details can be found in Supplemental Table I. We collected immunohistochemistry (IHC) data for ERα and PR for both pre- and post-treatment samples of 26 of 28 patients (Supplemental Figure 1A). No tumors were HER2-positive. Clinical marker levels (ERα and PR) did not differ between sample origin hospitals. We found no significant differences between pre- and post-tamoxifen treatment ERα levels (Wilcoxon rank-sum, *P* = 0.26) (Figure 1B, 1C). A trend was identified for higher PR levels after tamoxifen treatment, however it was not significant (Wilcoxon rank-sum, *P* = 0.08) (Figure 1B, 1C). Gene expression levels for the corresponding coding genes, Estrogen receptor 1 (ESR1) and progesterone receptor (PGR) were also not significantly different between pre- and post-treatment samples (data not shown). When investigating the cell proliferation marker Ki67 (MIB1), post-treatment samples had significantly lower gene expression (MKI67) levels (Wilcoxon rank-sum, *P* < 0.01) (Figure 1D). A similar significant trend was found when measuring Ki67 at the protein level with IHC (data not shown). Because IHC Ki67 score is difficult to interpret with substantial inter-observer variation [43], we chose to examine microarray gene expression levels of MKI67 as they are less subjective. In addition, we classified all our samples for two known molecular classifiers with links to outcome, MammaPrint and IntClust (Supplemental File 1).

**Table 1:** Patient characteristics

	Male	Premenopausal	Postmenopausal	
	*N* = 2	*N* = 14	*N* = 12	
Variable	No. (%)	No. (%)	No. (%)	P*
Year of diagnosis				0.004^a^
Mean	2011	2012	2010	
Range	2011 - 2011	2011 - 2013	2008 - 2012	
Age at diagnosis				<0.001^a^
Mean	63	47	62	
Range	52 - 73	41 - 54	52 - 79	
Treatment duration				0.189^a^
(days)				
Mean	26	22	18	
Range	25 - 26	9 - 45	8 - 35	
Tumor size by ultrasound (mm)				0.954^a^
Mean	20	13.6	13.8	
Range	18 - 22	0 - 27	0 - 30	
Lymph node status				0.108^b^
micrometastasis	0 (0.0)	3 (10.7)	1 (3.6)	
positive	1 (3.6)	0 (0.0)	3 (10.7)	
negative	1 (3.6)	11 (39.3)	7 (25.0)	
other	0 (0.0)	0 (0.0)	1 (3.6)	
Tumor histological grade				0.125^b^
1	1 (3.6)	5 (17.9)	4 (14.3)	
2	0 (0.0)	5 (17.9)	8 (28.6)	
3	1 (3.6)	4 (14.3)	0 (0.0)	
Tumor histology				0.209^b^
IDC	2 (7.1)	11 (39.3)	11 (39.3)	
ILC	0 (0.0)	3 (10.7)	0 (0.0)	
IDC + invasive carcinoma	0 (0.0)	0 (0.0)	1 (3.6)	

* *P*Tests are performed only on premenopausal and postmenopausal data;

a Wilcoxon-rank-sum-test;

b Pearson′s chi-squared test

### Tamoxifen reprograms ERα binding in tumors

**Figure 2:**
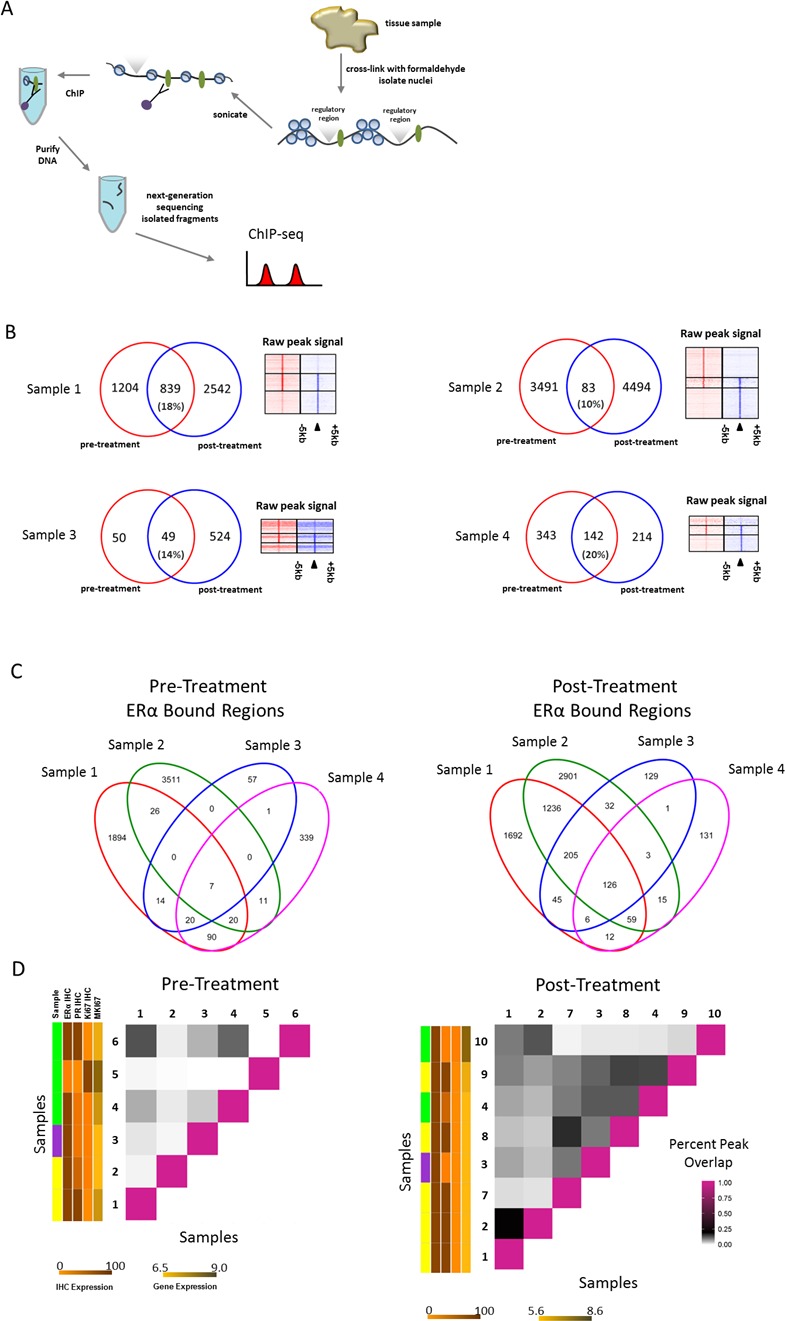
ERα binding events before and after neoadjuvant tamoxifen treatment **A.**, Schematic representation of ChIP-seq process. ERα bound to the DNA is depicted as green ovals; regulatory regions are depicted above the DNA strand as grey triangles. **B.**, Venn diagrams depicting the overlap of ERα bound regions (peaks) in both pre-(red) and post-(blue) treatment pairs. Heatmaps on the right show ERα binding events at the center of the peak and the surrounding ±5kb for the pre-treatment sample only peaks (top), overlapping peaks (middle) and post-treatment sample only peaks (bottom). **C.**, Venn diagrams depicting all overlapping peaks for pre- and post-treatment pair samples separately. **D.**, Matrix to visualize percent of overlapping peaks (of total peaks in each pair combination) for both pre- and post-treatment samples separately. Vertical colored side bars indicate patient menopausal-status (sample) and ERα IHC, PR IHC, Ki67 IHC and MKI67 values respectively. Patient menopausal-status is shown in yellow (post-menopausal), green (pre-menopausal) and purple (male). Separate scale bars are shown below the plot for expression levels corresponding to sidebars. IHC expression scale bar for both panels indicates percentage staining nuclei. Gene expression scale bar for both panels indicates normalized gene expression values.

Since tamoxifen is aimed to directly target ERα action, ERα functioning may also be affected on the genomic level by drug treatment. Therefore, we analyzed the chromatin binding profiles of ERα in 6 pre- and 8 post-treatment samples (4 treatment pairs) using ERα ChIP-seq (Figure 2A; Supplemental Figure 1A). We identified ERα bound genomic regions, as exemplified for two typical ERα regions in the human genome found at the enhancer regions proximal to the RARA and IGFBP4 loci (Supplemental Figure 2A). As expected, the top DNA binding motifs identified in both pre- and post-treatment were found to be the hormone nuclear receptor family with ESR1 as the top factor (Supplemental Figure 2B).

A relocation of ERα chromatin interactions to other sites was observed after tamoxifen treatment (Figure 2B, Supplemental File 2). Our laboratory [44] and others [26] previously reported ERα chromatin interaction profiles as highly heterogeneous between tumors. Consistent with previous findings, we found limited overlap of ERα binding patterns between two tumors prior to therapy (Figure 2C). Importantly, the pattern overlap was substantially increased after neoadjuvant tamoxifen therapy in treatment pairs (Figure 2C). To investigate this further in a quantitative fashion, the percent of overlapping ERα bound genomic regions between all samples of the total ERα bound genomic regions was calculated in each possible sample combination (within pre- or post-treatment condition) (Figure 2D). The average percent overlap in pre- and post-treatment samples increased from 3.0 to 7.9 with data ranging from 0.01% to 13% overlap among pre-treatment samples and 0.8% to 21% overlap among post-treatment samples. We identified a significant (Wilcoxon signed rank, *P* < 0.001) increase in overlap in genomic regions in the post-treatment group compared with the pre-treatment group.

Next, we characterized the genomic regions of the tamoxifen-induced synchronized sites (I) and pre-treatment unique sites (II) (Supplemental Figure 2C). In genomic location for both, the sites are typical of what is known of ERα binding in that they are found mostly in distal intergenic and intronic regions (Supplemental Figure 2D) [45]. In addition, the most common sequence motifs associated with both groups of sites were ESR1 and ESR2 (Supplemental Figure 2E). Similar findings are observed for pre-treatment unique sites. Next, we determined the nearest gene for each of the 126 ERα bound sites (I) within 20kb [46]. Using this definition, 96 genes are associated with these tamoxifen-synchronized sites. When examining these genes using Ingenuity Pathway Analysis (Qiagen), we find these genes involved in drug and lipid metabolism, estrogen receptor signaling and notably, EGF was identified as a potential upstream regulator. (Supplemental Figure 2F).

### ERα, PR and FOXA1 binding profiles in tumors and MCF7 cells reveal dynamic FOXA1 binding

**Figure 3:**
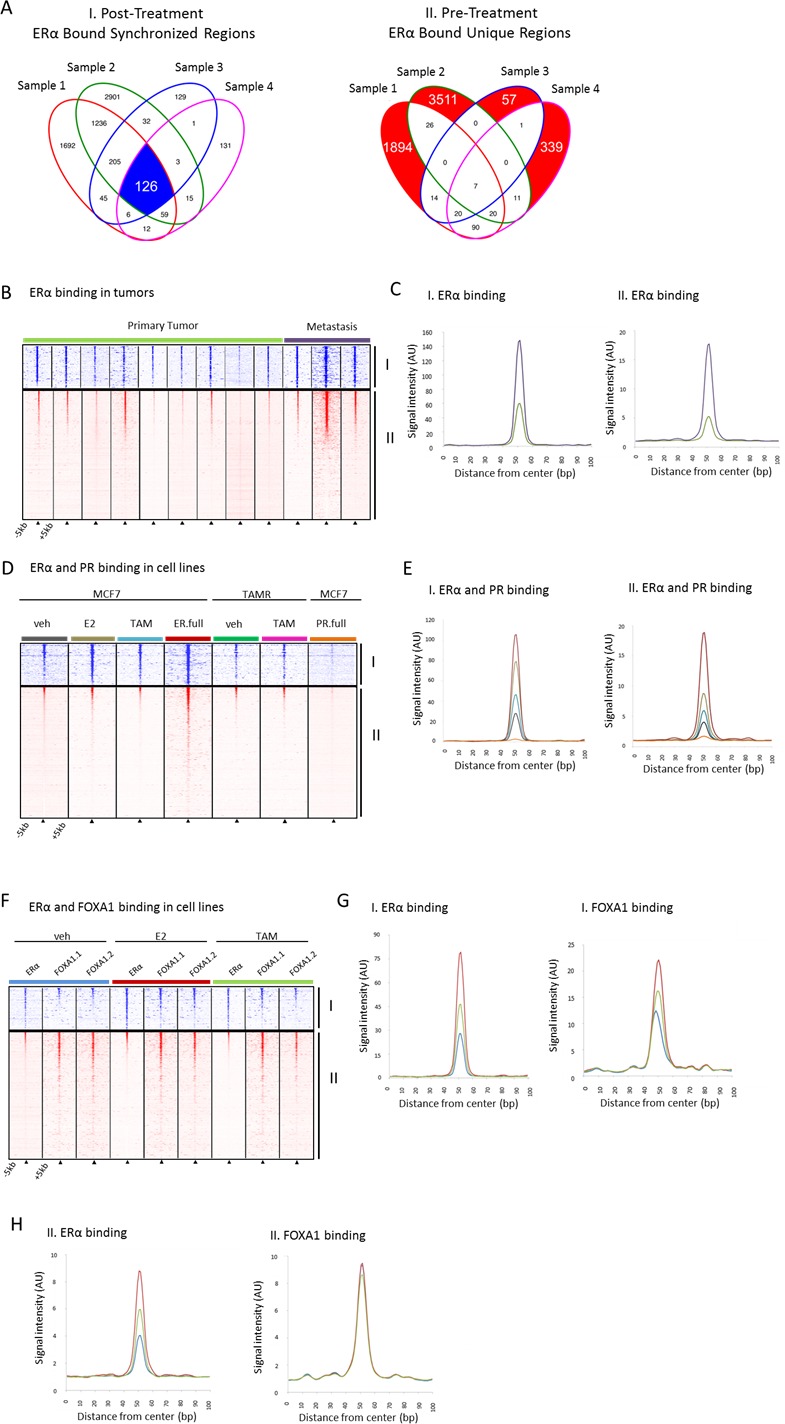
Binding profiles of 126 tamoxifen-synchronized regions (I) and unique pre-treatment regions (II) **A.**, Venn diagrams depicting overlapping peaks (paired samples) for both post-treatment tamoxifen synchronized sites (I) and unique pre-treatment sites (II). **B.**, Heatmap showing binding peak intensity for ERα binding events in I and II sites (± 5kb) in primary tumors (green) and metastases (purple).**C.**, Normalized average signal intensity of ERα binding events from panel B. Line colors match B. **D.**, Heatmap showing binding peak intensity for C for ERα binding events in I and II sites (± 5kb) in MCF7 cell lines deprived of hormones for three days and then given vehicle (grey), estradiol ((E2), brown), tamoxifen ((TAM), blue) and full medium ((ER.full), red). PR binding in MCR7 cell lines deprived of hormones for three days and then given full medium is shown in orange (PR.full). TAMR cell lines deprived of hormones for three days and then given vehicle (green) and TAM (purple) are also depicted. **E.**, Normalized average signal intensity of ERα binding events from D. Line colors match D. **F.**, Heatmap showing binding peak intensity for ERα and FOXA1 binding events in I and II sites (± 5kb) in MCF7 cell lines deprived of hormones for three days and then given vehicle (blue), E2 (red) and TAM (green). **G.**, **H.**, Normalized average signal intensity of ERα (left) and FOXA1 (right) binding events from panel F. Line colors match F.

**Figure 4:**
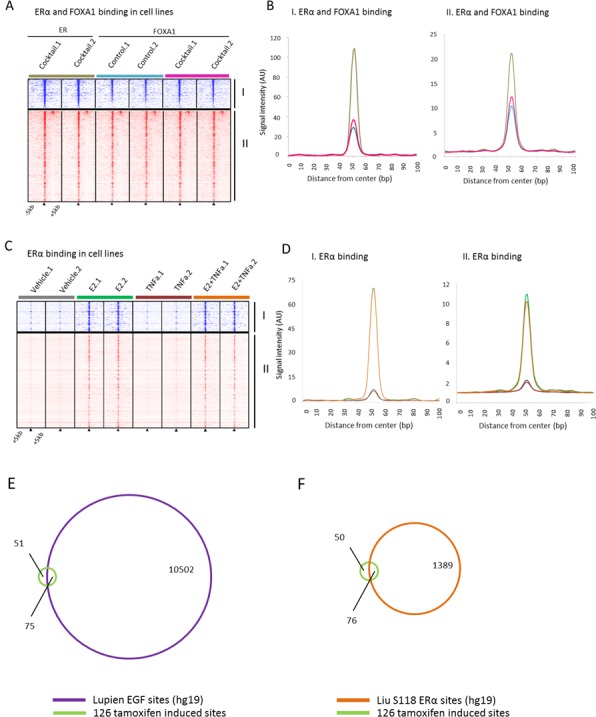
Binding profiles of 126 tamoxifen synchronized regions (I) and unique pre-treatment regions (II) under mitogen conditions **A.**, Heatmap showing binding peak intensity for ERα/FOXA1 binding events in I and II sites (± 5kb) in MCF7 cells, cultured under control conditions (blue) or in presence of a mitogen cocktail containing EGF, IGF-1, TNFα and IL-6 (brown for ERα and pink for FOXA1). **B.**, Normalized average signal intensity of ERα/FOXA1 binding events from panel B. Line colors match B. **C.**, Heatmap showing binding peak intensity for ERα binding events in I and II sites (± 5kb) in MCF7 cell lines deprived of hormones for three days and then given vehicle (brown), E2 (green), TNFα (burgundy), or the combination of both (orange). **D.**, Normalized average signal intensity of ERα binding events from panel C. Line colors match D. **E.**, Venn diagram depicting overlap of the 126 tamoxifen-induced bindings sites with EGF-induced ERα sites. **F.**, Venn diagram depicting the overlap of tamoxifen-induced binding sites with estradiol induced S118-ER sites.

ERα binding and activity in breast cancer is regulated and controlled by various other factors such as FOXA1, a pioneering factor required for binding of ERα [47 52] and PR, a modulator of ERα action in breast cancer [53]. When examining the 126 ERα bound sites synchronized by tamoxifen (Figure 3A (I)) in publicly available ChIP-seq data for tumors, we found the average relative signal intensity at these sites is higher in metastatic samples compared with primary tumors samples (ERα/PR positive) (Figure 3B, 3C). A similar pattern is seen in the unique pre-treatment sites (Figure 3A (II)) but with much less overall signal intensity (Figure 3B, 3C). In addition, in MCF7 cells we observed a similar pattern in response to tamoxifen or estradiol in ERα binding at these sites (Figure 4D, 4E). No PR binding was observed (Figure 3D, 3E) under full medium conditions for either the 126 synchronized sites or the unique pre-treatment sites. FOXA1 chromatin binding is described as independent of ERα action and irresponsive to hormonal stimuli [50, 51]. However, FOXA1 chromatin binding at these 126 sites was clearly induced by both tamoxifen and estradiol, accompanied by ERα binding. These data imply a unique feature of the 126 tamoxifen-synchronized ERα sites, hallmarked by dynamic FOXA1 action at these sites.

### ERα, FOXA1 binding profiles in MCF7 cells reveal potential mechanistic players

To better understand a possible mechanism of ERα and FOXA1 reprogramming to these sites after addition of tamoxifen, we investigated publically available ERα and FOXA1 ChIP-seq data from MCF7 cells treated with specific mitogens and/or growth factors [50]. Data from Hurtado and colleagues indicate the overlap of ERα binding sites for both estradiol and tamoxifen treated MCF7 cells is substantial. Furthermore, the proportion of tamoxifen only binding sites is very small (7%) [50]. Within our paired tumor data, we show that on average 56.3% of the total sites are post-tamoxifen treatment only binding sites (Figure 2B).

To examine a potential mechanism involved in synchronizing ERα to these sites based on known regulators of ERα binding, we investigated the Ross-Innes mitogen cocktail data, which contains EGF, IGF-1, TNFα and IL-6 [26], the Franco TNFα data [54] and the Lupien EGF data [52] at the tamoxifen synchronized and pre-treatment unique sites. The mitogen cocktail significantly induced FOXA1 binding at these sites (Wilcoxon signed rank, *P* < 0.0001). (Figure 4A, 4B). In ERα binding data where estradiol, TNFα or a combination of the two were added, we observed no increased FOXA1 binding by TNFα (Figure 4C, 4D). Combined, these data rule out TNFα alone as a key regulator of FOXA1 dynamics at these sites, leading us to investigate EGF as it was implicated in our own Ingenuity Pathway Analysis results. ChIP-chip data of EGF-stimulated ERα binding sites show high overlap (60%) with our tamoxifen induced sites (Figure 4E, Supplemental File 3). We found the enrichment for these sites within the pre-defined universe of known ERα binding sites to be significant (Fisher's hypergeometric test, *P* < 1.10E-15) (Supplemental File 3) indicating the high proportion of overlap of tamoxifen induced sites are biologically significant. In addition, we observed comparable results when looking at the overlap of the tamoxifen induced sites with serine 118 ERα DNA binding sites induced by estradiol (Figure 4F) [55]. Cumulatively, these data implicate EGF as a potential upstream regulator of FOXA1-induced chromatin binding at the 126 sites, facilitating ERα action at these sites in tumors post tamoxifen exposure.

### DNA copy number profiling and alternate allele frequency analysis reveal little variation between replicates and/or treatment condition

We observed ERα binds to different sites after neoadjuvant tamoxifen treatment (Figure 2D). We questioned if the change in ERα binding is due to genomic changes within the tumor population or re-targeting of ERα. Notably, interaction profiles [56] and overall library read numbers [23] have been shown to be reproducible within replicate tumor samples. To determine the potential impact of intra-tumor genomic variability, we examined DNA copy number profiles (example in Supplemental Figure 3A) of 3 pairs of replicate tumor samples that were ChIP-sequenced twice. Replicate samples were sectioned from the same tumor at a different time with at least 5 cell layers (25 to 50μm) between replicates. DNA copy number data were obtained from the off-target read in ChIP-seq experiments using the CopywriteR method [30] (Supplemental Figure 1A) and data were subsequently segmented with circular binary segmentation. We did not identify any substantial changes in the DNA copy number profiles between sample replicates. (Supplemental Figure 3B). Unsupervised hierarchical cluster analysis on all the copy number data (20kb region bins, non-segmented) found most replicates cluster together with the exception of sample pair D (*n* = 12) (Supplemental Figure 3C). To quantify this further we calculated *p*-values (100 indicates a *p*-value < 0.00001) for the clustering based on multiscale bootstrap resampling and bootstrap resampling using the R package ‘pvclust’. (Supplemental Figure 3D) The *p*-values indicate the cluster analysis is highly supported by the data illustrating the low intra-tumor genomic variability of the samples as measured by DNA copy number. In addition, in our tumor ChIP-seq replicate data, we observed high correlation of read counts in known ERα binding regions [26] (Spearman's *rho* 0.40 and 0.41) within replicate sample pairs and low correlation in 3 un-related samples to one of the replicate samples (Spearman's *rho*, < 0.10) (Supplemental Figure 3E).

To examine whether there is evidence that neoadjuvant tamoxifen treatment selects for a specific tumor sub-clone we also analyzed the copy number profiles of 4 treatment pairs, both pre- and post-treatment (Supplemental Figure 1A). Interestingly, we found no major differences in the copy number profiles between sample timing (Supplemental Figure 3F) suggesting treatment does not affect the overall DNA makeup in our samples. Unsupervised hierarchical cluster analysis of all the copy number data (non-segmented 20kb region bins) showed pre- and post-treatment samples cluster together, supporting the conclusion that neoadjuvant tamoxifen treatment does not confer DNA copy number changes in the samples (*n* = 8) (Supplemental Figure 3F, 3G) or select for a small sub-clone with a different DNA copy number pattern. The resultant *p*-values indicate our cluster analysis is strongly supported by the data (Supplemental Figure 3H).

To quantitatively investigate the relative contribution of tumor DNA between treatment groups we called heterozygous single-nucleotide variants (SNVs) in both pre- and post-treatment ChIP-seq pairs. Assuming 100% tumor DNA in our samples, each heterozygous SNV should have a ratio of 50/50 for alternate/reference reads (alternate allele frequency). Changes in the tumor content between treatment conditions should be evident in alternate allele frequency. We required each SNV to have at least 10 reads covering the alternate and reference allele for analysis. A scatterplot shows the alternate and reference allele counts in each SNV called (Supplemental Figure 3I). The distribution of alternate allele frequency between treatment groups was not significantly different (Wilcoxon signed rank, *P* = 0.76). Furthermore, we observed no significant difference (Wilcoxon rank sum, *P* = 0.37) between pre- and post-treatment alternate allele frequency in paired analysis taking into account only variants identified in both cases of the treatment pair (Supplemental Figure 3J). These findings support our conclusions from unsupervised hierarchical clustering of DNA copy number that pre- and post-treatment changes are not detectable at the level of DNA.

### Pre- and post-treatment differential gene expression

Since ERα is a transcriptional regulator, we next examined differences between pre- and post-treatment samples at the gene expression level for 20 pre-treatment and 26 post-treatment samples (19 treatment pairs) using microarray technology on FFPE samples (Supplemental Figure 1A). We used ANOVA analysis to determine the most differentially expressed genes between the two classes, pre- and post-treatment, and visualized the results with unsupervised hierarchical clustering (Supplemental Figure 4A). Using the top variable genes from the dataset (variance > 1 across samples) we found 189 genes to be differentially expressed (FDR < 0.001 and fold-change > 2, Supplemental Figure 4A). To identify key biological processes regulated by the differentially expressed genes, we used Ingenuity Pathway Analysis. The most upregulated genes in post-treatment samples are found in the adipogenesis pathway and are involved in LXR/RXR activation and include FBJ murine osteosarcoma viral oncogene homolog (FOS), nuclear receptor subfamily 4, group A, member 1 (NR4A1; NUR77) and dual specificity phosphatase 1 (DUSP1) (Supplemental Figure 4B, Supplemental File 4). Diseases such as cancer and neurological/cardiovascular disease along with biological functions such as cellular movement and apoptosis were associated closely with the genes upregulated after treatment (Supplemental Figure 4B). Interestingly, the most downregulated genes in post-treatment samples compared with pre-treatment samples were associated with immune response, such as REX1 RNA exonuclease 1 homolog (REXO1L1P) (Supplemental Figure 4B, Supplemental File 4). Genes that were downregulated after treatment were also associated with diseases such as cancer, endocrine system disorders and breast carcinoma.

In addition, we found MKI67 gene expression was significantly reduced in the post-treatment samples (Wilcoxon rank-sum, *P* = 0.003) (Figure 1D). To examine the relationship between MKI67 levels and proliferation we determined the gene expression proliferation module scores based on published modules AURKA and CIN70 [34] and calculated the percent change as described in the module score between pre- and post-treatment. The percent change in known proliferation modules is highly correlated with percent change in MKI67 in our cohort (Supplemental Figure 4C, 4D). Notably, we found the pre-treatment gene expression of proliferation module genes was significantly more variable than in the post-treatment condition (Wilcoxon rank-sum, *P* < 0.001), suggesting expression changes in proliferation module genes are driven by the tamoxifen treatment (Supplemental Figure 4E).

### Genes associated with 126 tamoxifen induced synchronized sites differentiate patient outcome

**Figure 5:**
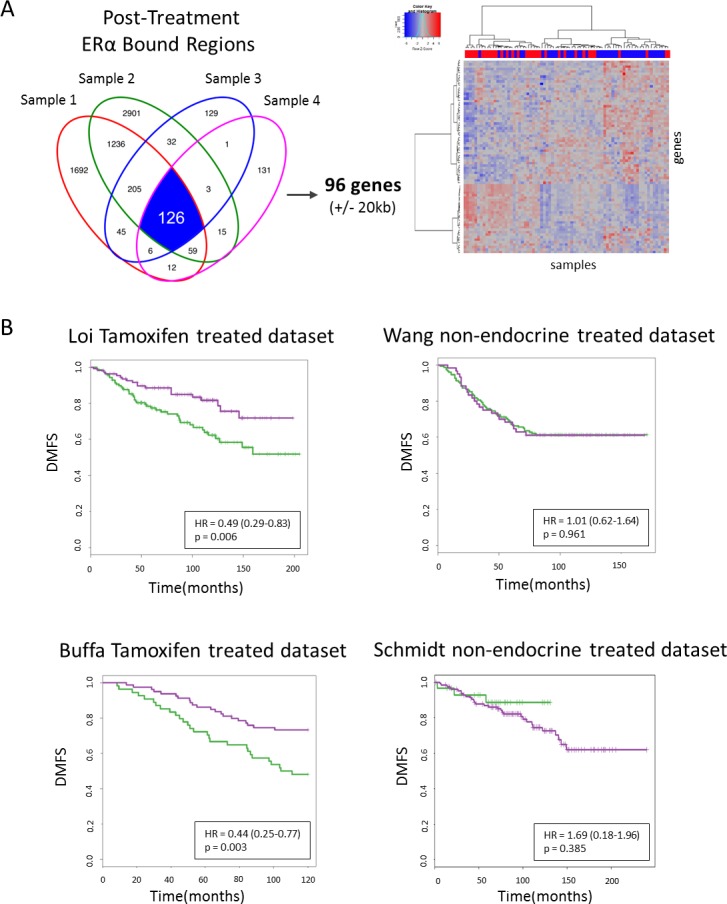
96 genes survival analyses **A.**, Venn diagram depicting overlapping peaks (paired samples) for post-treatment tamoxifen synchronized sites (I) and an unsupervised hierarchical clustering heatmap depicting gene expression in our series of those genes. Top row indicates pre-treatment samples (red) and post-treatment samples (blue). **B.**, Kaplan Meier survival curves of distant metastasis-free survival for tamoxifen treated datasets (left panels) and non-endocrine therapy treated datasets (right panels).

After identifying the 96 genes associated with the 126 tamoxifen-induced sites (±20kb from the nearest transcription start site) (Figure 5A), we first determined these genes strongly separate pre- and post-treatment samples (Figure 5B). Next, we wished to examine their capacity to predict patient outcome using distant metastasis free survival (DMFS). For this, we used publically available gene expression and clinical data from two data sources, (i) tamoxifen treated [38, 39], *n* = 250, *n* = 134, respectively (ii) and non-endocrine therapy treated [40, 41], *n* = 209, *n* = 158, respectively (Figure 5A, 5B). For tamoxifen-treated patients (*n* = 250, [38]), the 96 genes are capable of differentiating good *versus* poor outcome for the unsupervised hierarchically defined groups using DMFS (hazard ratio (HR) = 0.49, *P* = 0.006). This result was validated in a second cohort (HR = 0.44, *P* = 0.003; *n* = 134, [39]). Based on these 96 genes, no significant differential outcome was found in the two ERα positive cohorts of non-endocrine therapy treated patients [40, 41] (HR = 1.01, *P* = 0.961; HR = 1.69, *P* = 0.385, respectively), indicating the genes are not prognostic in nature (Figure 5B).

## DISCUSSION

In breast cancer, biomarker discovery is classically performed on treatment-naive tissue samples from surgery. While this is a logical choice from a clinical perspective, it may not be the most ideal setting for predicting response to therapy. This notion was stressed by the recent discovery of specific ESR1 (the gene that encodes for ERα) mutations in relapsed tumor tissue, after having received adjuvant tamoxifen or aromatase inhibitor therapy [57, 58]. These findings illustrate that ESR1 mutations do occur in breast cancer and that, more importantly, clinically relevant data may only be discovered when the clinicopathological or molecular assessment is performed after treatment pressure has occurred.

Inter-tumor heterogeneity of breast cancer, both histopathological and molecular, is well documented [2 9] and recent findings indicate the tumor itself is frequently genomically heterogeneous [59, 60]. Using DNA copy number profiling we did not observe substantial differences in biological replicates from the same tumor as Wang *et al*. also previously reported using single cell whole genome sequencing [61]. When analyzing biological replicate ChIP-seq samples we found a high correlation within pairs with respect to read count in known ERα binding regions compared with un-related samples indicating the changes observed in ERα binding are induced by neoadjuvant tamoxifen treatment and not inherent to intra-tumor variability in ERα binding. In addition, we found no evidence that treatment induces differences at the copy number or single nucleotide level suggesting that tamoxifen treatment did not select for certain sub-clones, defined by copy number aberrations and alternate allele frequency. Moreover, when examining the gene expression data alone (Mammaprint, Supplemental File 1) [7] and the integrated DNA copy number and gene expression data using IntClust classifications (Supplemental Data File 1) [36, 37] we found few changes between classes and an enrichment for the Luminal/ERα-positive subtypes. We are unable to rule out the possibility that there are differences in the DNA at the global level of single nucleotide variations with these data.

When examining the 126 tamoxifen-induced ERα bound sites in additional datasets we found a higher level of binding in metastatic samples compared with primary tumor samples from breast cancer patients. The metastatic patients from this series (Ross-Innes *et al*. 2012), failed to respond to endocrine therapy so we can categorize these samples as comparable to the post-treatment situation in our series. In this respect, our findings recapitulate the original observation of higher ERα binding site signal intensity in metastases relative to primary tumors. In addition, we found in MCF7 cells that PR binding is not present in the 126 tamoxifen synchronized sites indicating the binding at these regions is not modulated by PR. As expected, tamoxifen, estradiol and full medium conditions resulted in more binding at these sites than vehicle conditions. Interestingly, FOXA1 binding at these sites had a similar pattern in response to tamoxifen and estradiol as ERα, showing FOXA1 is dynamic at these sites while the vast majority of FOXA1 sites are not [50]. Dynamic FOXA1 sites are indicative of outcome [26] and affected by growth factors [26, 52, 54].

It has been previously reported that tamoxifen treatment induces ERα chromatin binding in MCF7 luminal breast cancer models at the same genomic sites as estradiol treatment [50]. Importantly, these data are not in line with our observation *in vivo* in 4 independent tumors treatment pairs, where tamoxifen treatment resulted in a relocation of ERα chromatin interactions to other sites. The overlap of ERα binding sites of estradiol and tamoxifen conditions for MCF7 cells is substantial [50]. We observed in our paired tumor data a far greater proportion of post-tamoxifen treatment only sites (56.3%) in contrast to what is known from cell-line data (7%). Ingenuity Pathway Analysis indicated EGF to be an upstream regulator of relocation of ERα to these sites. We found a substantial proportion of overlap of these synchronized sites with EGF-induced sites in MCF7 cells further implicating EGF as a regulator by which ERα is relocated to these sites after tamoxifen. Importantly, we also observed at these sites that FOXA1 is responsive to tamoxifen and estradiol in tumors. With this, we suggest a possible mechanism by which ERα and FOXA1 are reprogrammed to these sites after tamoxifen mediated by EGF. Also, our findings that FOXA1 is dynamic in these regions suggest that this specific subset of FOXA1 binding sites may be dependent on ERα.

Interestingly, our findings in both tamoxifen treated cohorts indicate genes associated with tamoxifen-induced ERα-synchronization are capable of specifically identifying breast cancer patients, who may not benefit from tamoxifen treatment. Furthermore, the observations from non-endocrine therapy treated cohorts suggest these genes are not associated with prognosis. These data are highly clinically relevant as around 50% of patients that receive tamoxifen experience a recurrence. With the 96-gene classifier reported here, 57% and 63% of patients are predicted to be tamoxifen resistant in the two tamoxifen-treated cohorts examined. Although these findings were reproducible between multiple publically available cohorts, they must be validated in the context of a prospective randomized clinical trial in order to examine the robustness of the gene set's capacity to predict tamoxifen benefit. In addition, one would like to adjust the hazard ratios reported in this study for standard prognostic factors, such as age, histological grade *etc*. Unfortunately, for all datasets these additional clinico-pathological data are not publicly available.

A portion of ERα-positive breast cancers also express EGFR (see review by Osborne and Schiff and references therein) [62, 63]. We have observed in our own data an indication that EGF is an upstream regulator at the 126 tamoxifen-synchronized sites. From our exploration of genes associated with these sites, we determined a subset of patients do not respond well to tamoxifen treatment. These patients may benefit from a combined therapy of tamoxifen and cetuximab, an anti-EGFR antibody, or an EGFR tyrosine kinase inhibitor. Two studies in metastatic breast cancer patients explored either tamoxifen [64] or anastrozole [65] with or without gefitinib and found a numerical advantage for the addition of gefinitib to endocrine therapy regarding clinical benefit rate and progression-free survival, but only in endocrine-therapy naïve patients, or patients who had come off adjuvant tamoxifen at least 12 months before recurrence [64, 65]. In the neoadjuvant setting postmenopausal patients with EGFR-positive, and ERα-positive breast cancer were randomized for gefitinib (EGFR tyrosine kinase inhibitor) alone or gefitinib plus anastrazole, an aromatase inhibitor. Both the single and combination agents reduced tumor size [63], and induced Ki67 downregulation as well as phospho-EGFR. Taken together, these data all support the existence of a subgroup of ERα-positive breast cancers that become endocrine therapy resistant through activation of the EGFR signaling pathway, which may be mediated by a dynamic FOXA1 DNA binding landscape. The current data will help to further define those patients that will derive benefit from the addition of an EGFR inhibitor to endocrine therapy.

Although neoadjuvant tamoxifen treatment times in this study were relatively short and varied (Table I), we did observe similar trends in transcriptomic changes in the 7 patients treated less than 2 weeks (Supplemental Figure 5A, 5B). We did not examine the DNA copy number or ChIP-seq data for these samples as only one patient in this short-treatment subgroup had paired data available. Overall, we observed a significant synchronization in the ERα/chromatin binding regions in the post-treatment samples compared with pre-treatment samples.

ERα is a key transcription factor in cellular proliferation and importantly we found gene expression proliferation module signatures are significantly less variable in the post-treatment samples indicating a synchronization of the gene expression after treatment. In addition, changes in gene expression modules are significantly correlated with changes in MKI67 levels indicating the power of MKI67 levels to detect changes in proliferation. The gene expression changes coupled with the overall stability of the DNA copy number profiles between treatment conditions suggests transcriptional alterations are mediated by ERα/chromatin binding induced changes conferred by neoadjuvant tamoxifen treatment.

We present the first comprehensive assessment of DNA copy number, gene expression patterns and ERα/chromatin profiles at two different time points of breast cancer therapy: before and after neoadjuvant tamoxifen treatment. The patient series investigated in this work is relatively small, however utilizing the power of paired treatment samples we are able to determine molecular changes conferred by neoadjuvant tamoxifen treatment. Large-scale alterations of ERα action were observed due to therapy, resulting in a substantial synchronization of ERα/chromatin binding and gene expression patterns between patients. We uncovered evidence these synchronized sites may be important in breast cancer outcome as they show more binding in metastatic samples *versus* primary tumor samples. In addition, we found FOXA1 binding at these sites to be dynamic in response to estradiol and tamoxifen. Binding profiles at these sites implicate EGF as a potential regulator of these sites. FOXA1 dynamic binding is linked to outcome and critically, we identify genes associated with tamoxifen-induced sites to be capable of differentiating patients for tamoxifen benefit.

With this, we illustrate that hormonal therapy in breast cancer reprograms the genomic behavior of the drug target, ERα, and consequently affects downstream proliferation gene programs linked to patient outcome. Due to the direct effects of therapeutics on transcription factor behavior and transcriptional output, biomarker discovery studies may be further facilitated by performing such studies after neoadjuvant drug exposure.

## MATERIALS AND METHODS

### Patients and characteristics

ERα-positive breast cancer patients were recruited as part of the ongoing AFTER study (Anastrozole, Fulvestrant or Tamoxifen Exposure Response in molecular profile, ClinicalTrials.gov #NCT00738777) at two Dutch hospitals (Netherlands Cancer Institute-Antoni van Leeuwenhoek (NKI-AVL; Amsterdam) and Radboud University Medical Center (RadboudUMC; Nijmegen). Patient accrual occurred between August 2008 and February 2013. Local medical ethical authorities at both centers approved of the collection protocols. Pre-menopausal and male patients were treated with tamoxifen. Post-menopausal patients were assigned randomly to tamoxifen, anastrozole or fulvestrant therapy. Among them, only data from tamoxifen treated patients were analyzed (Supplemental Figure 1B). Patients were eligible if they had invasive, non-inflammatory breast cancer and were treated with hormonal therapy if the tumor was hormone receptor-positive by immunohistochemical (IHC) staining at diagnosis. Patients were excluded if they had multi-centric or metastatic breast cancer or if they received hormone replacement therapy in the previous 12 months. Patients were treated during the time between core-needle biopsy for diagnosis and surgery (typically 2-6 weeks). In a normal setting tamoxifen can take up to 8 weeks to reach therapeutic, steady-state levels in the blood plasma [21, 22]. Based on pharmacokinetics studies by Fabian *et al*, we chose a loading dose of 40mg orally, twice daily during 7 days with follow-up standard dosage of 20mg orally once daily to be able to reach steady state levels within 2 weeks [21].

We attempted to collect both pre-treatment and post-treatment fresh-frozen (FF) and formalin-fixed, paraffin-embedded (FFPE) material for each patient (Supplemental Figure 1A). Sample fixation for each assay is listed in the methods details below. Ten serial sections of 5μm each were taken from both the core-needle biopsy (14 gauge) and primary tumor FFPE material for analyses. Pre-treatment FF material was in the form of core-needle biopsy made up of 10-15 serial sections of 30μm each. Post-treatment FF material was sectioned in 30 serial sections of 30μm each from the primary tumor taken at the time of surgery. All replicate experiments are from additional sections taken from the same tumor.

### Immunohistochemical staining and assessment

Immunohistochemistry of samples was performed on a BenchMark Ultra autostainer (Ventana Medical Systems) for ERα, Progesterone Receptor (PR) and Receptor Tyrosine-Protein Kinase erbB2 (HER2). Briefly, paraffin sections were cut at 3μm, heated at 75°C for 28 minutes and deparaffinized in the instrument with EZ prep solution (Ventana Medical Systems). Heat-induced antigen retrieval was carried out using Cell Conditioning 1 (CC1, Ventana Medical Systems) for 36 minutes at 95°C (ERα, PR and HER2) or 32 minutes at 95°C (Ki-67). ERα was detected using clone SP1 (ready-to-use dispenser, 32 minutes room temperature (RT), Roche), PR with clone 1E2 (ready-to-use dispenser, 32 minutes RT, Roche), HER2 with clone 4B5 (ready-to-use dispenser, 12 minutes RT, Roche) and Ki67 detection using clone MIB1 (1:250 dilution, 32 minutes RT, DAKO). Bound antibody (ERα, PR and HER2) was detected using the UltraView Universal DAB Detection Kit (Ventana Medical Systems), while detection for Ki67 was visualized using the OptiView DAB Detection Kit (Ventana Medical Systems). Slides were counterstained with hematoxylin. ERα/PR/HER2/Ki67 scoring was performed on whole slides by a single pathologist (JW) blinded to patient status. For ERα, PR and Ki67 the percent of positive tumor nuclei was determined. For ERα/PR, 10% was used as a cut-off for positive according to current standard European guidelines. HER2 scoring was performed on a 0 to 3+ intensity scale examined in the nuclei (0, negative staining; 1+, weak staining; 2+ moderate staining, 3+, strong staining). There were no ≥ 2+ HER2 samples in the cohort.

### Chromatin immunoprecipitation and sequencing

Chromatin immunoprecipitations (ChIP) were carried out on FF material as previously described [23] using 5μg of ERα antibody (SC-543; SantaCruz Biotechnology) and 50μl Dynabeads. Subsequently, Illumina-indexed libraries were constructed for each sample using the ERα ChIP DNA (ChIP-seq) (Figure 2A). Indexed DNA from 9 11 samples was equimolarly pooled sequenced on a single Illumina HiSeq2000 flowcell lane. Single-end 50bp reads were generated for each sample. Raw sequence data were aligned to the human genome (Ensembl 37) using Burrows-Wheeler Aligner (BWA) (mapping quality ≥ 20, duplicate reads removed). As control, input chromatin was used. For each treatment category, 6-10 control samples were pooled equimolarly together to create a meta-pool of control input material. This meta-pool material was used as a reference sample for further analysis.

### Analysis of ChIP-sequencing

To identify enriched genomic regions we used two peak callers, MACS [24] and DFilter [25]. We ran MACS using the default parameters with the exception of a more significant *p*-value threshold at 1.00e-7. DFilter settings were default for transcription factor detection. The intersection of the peaks from both peak callers was used for the final list of enriched regions (peaks) for each sample. Treatment conditions were analyzed separately for peaks where the corresponding treatment control input was used as reference.

We used the R Bioconductor ‘DiffBind’ package [26] to generate Venn diagrams of peaks for samples to determine a core set of binding events (identified in at least 2 of the samples in the set). This core set of binding events was used to identify the top DNA binding motifs using SeqPos from the Galaxy Cistrome package (http://cistrome.org/ap/) with z-score threshold set at −3.09, which corresponds to a *p*-value of 0.001 [27]. Other binding events were analyzed for DNA binding motifs using SeqPos from the Galaxy Cistrome package in the same manner. In addition, CEAS analysis was used from the Galaxy Cistrome package to characterize the genomic regions at binding sites.

To determine the intra-sample reproducibility of the ChIP-seq data we performed ERα ChIP-seq replicates on the same tumor sample using the same conditions. Additional data were analyzed for 3 supplementary ERα positive samples (not in the AFTER study) of similar overall read number. Read counts in 2,262 known non-overlapping ERα binding regions [26] were determined from the ChIP-seq alignment files using BEDTools coverageBed [28]. A correlation matrix of the read count data for all 5 samples (2 replicate pairs, 3 un-related) was then generated using the ‘cor’ function in R version 3.1.2. To determine the alternate allele frequency of variants in ChIP-seq data variants were called in ChIP-seq data of 4 treatment pairs using BWA, BCFTools and SAMTools. Only heterozygous single nucleotide variants with ≥10 alternate reads and ≥10 reference reads were chosen for further analysis. Alternate allele frequency is defined as the DP4 (SAMTools) alternate allele count divided by the DP4 total reads count at each SNV.

For analysis of public ChIP-seq datasets, raw sequence data were downloaded from GEO (GSE25315, GSE25316, GSE32222, GSE68355) and aligned and MQ filtered as above. We used SeqMINER version 1.3.3 [29] to visualize the aligned data at regions of interest (bed format).

### DNA copy number profiling

We employed the CopywriteR method [30] to detect DNA copy number from ‘off-target’ sequence reads taken from the ChIP-seq data without a reference sample. Circular binary segmentation was used to segment the data as previously described [30, 31].

### RNA isolations, microarray hybridization and analysis

Total RNA was isolated from formalin fixed paraffin embedded tissue as described previously [32]. After DNase treatment samples were purified using the Qiagen RNeasy FFPE kit. Total RNA (50ng) was reversed transcribed, amplified (Rubicon; C-WTA kit C) labeled with Cy3 (Genomic DNA enzymatic Labeling kit; Agilent Technologies) and subsequently purified (Amicon ultra 30kDa filters). Cy3-labeled cDNA was hybridized to custom full genome arrays—array design based on Agilent Catalog #G2514F—at 65°C for 17 hours and subsequently washed. Arrays were scanned with a dual laser scanner (Agilent Technologies). Image analysis of the scanned arrays was performed to quantify fluorescent intensities using Feature Extraction software version 9.5 (Agilent Technologies).

Feature signal intensities were processed, imputation of missing values and summarization of all genes with multiple probes was performed as previously described [33]. Visualization of the data was performed with Partek Genomics Suite (Partek) using Hierarchical Clustering (Pearson Dissimilarity, average linkage) with centered data. Top variable genes were selected based on variance across samples ( > 1). An ANOVA analysis was used to determine differentially expressed genes between treatment conditions of the top variable genes (Partek). Significant genes were selected univariately with FDR < 0.001 and the log2 space with fold change > 2.

To examine the relationship between published proliferation gene expression module scores and MKI67 gene expression levels we calculated the AURKA and CIN70 proliferation gene expression module scores (containing proliferation-associated genes (PAGs)) as described in the work by Martin and Dowsett and colleagues [34]. The percent change as described previously [34] between pre- and post-treatment gene expression module scores and MKI67 gene expression values were calculated and visualized with a scatterplot. In addition, MammaPrint^®^ scores were calculated [35].

### DNA copy number and gene expression integration

We used the R package ‘iC10’ to implement the classifier, IntClust to integrate and classify the samples into the 10 IntClust groups using DNA copy number and gene expression data [36, 37]. Classification was carried out as described previously using default settings [36].

### Statistical analysis

All statistical analyses were carried out in R version 3.1.2 (http://www.R-project.org) including all patients (pre-/post-menopausal and male) unless otherwise noted. Strength of associations between continuous variables was calculated with the Wilcoxon test, either Wilcoxon-rank sum (paired data) or Wilcoxon signed rank (non-paired data). For statistical analysis of categorical IntClust data, Pearson's chi-square test was used with simulated *p*-value based on 2000 replicates. For examining the uncertainty in DNA copy number hierarchical analysis we used the R package ‘pvclust’ (distance = correlation, clustering = Ward; number of iterations = 1000). Briefly, *p*-values were calculated *via* two methods, AU (approximately unbiased) and BP (Bootstrap probability). The AU method computes using multiscale bootstrap resampling and the BP method computes by normal bootstrap resampling. The resultant clusters were depicted visually as dendrograms with AU and BP values shown above the branch. To examine the relationship between the percent change in MKI67 gene expression and gene expression module percent change Spearman's *rho* was calculated. In addition, ChIP-seq sample replicate correlation using read counts in known ERα binding regions [26] was examined by Spearman's *rho*.

For survival analysis, normalized gene expression datasets were downloaded from GEO: GSE6532 [38], GSE22219 [39], GSE2034 [40] and GSE1121 [41]. From the tamoxifen treated datasets [38, 39], ERα positive, tamoxifen treated patients were selected, respectively (*n* = 250, *n* = 134). From the non-endocrine datasets [40, 41], ERα positive untreated patients were selected, respectively (*n* = 209, *n* = 158). ERα status for the Schmidt *et al.* dataset was determined as described previously [42]. For each dataset, we selected all probes matching the 96 genes associated with 126 tamoxifen-synchronized sites (disregarding genes not present on the array). The patients were then stratified into two groups with the selected gene expression data using unsupervised hierarchical Ward linkage clustering with Pearson correlation distance. Following that we calculated the hazard ratio to determine significant difference in distant metastasis-free survival (DMFS) between the two groups and Kaplan Meier graphs were constructed to visualize the data.

## SUPPLEMENTARY MATERIAL


