# Supplementary material for: Neoadjuvant tamoxifen synchronizes ERα binding and gene expression profiles related to outcome and proliferation
Source: Oncotarget. 2016 Apr 25;7(23):33901–18. doi: 10.18632/oncotarget.8983 (PMC5085127; doi:10.18632/oncotarget.8983)
Supplement: Supplementary file 1 [file oncotarget-07-33901-s001.pdf]

Neoadjuvant tamoxifen synchronizes ERα binding and gene expression profiles related to outcome and proliferation

Supplementary Material

TABLE OF CONTENTS

Supplemental Table 1……………………………………………………………S2

Supplemental Table 2……………………………………………………………S3

Supplemental Figure 1…………………………………………………………..S4

Supplemental Figure 2…………………………………………………………S5-S6

Supplemental Figure 3…………………………………………………………….S7

Supplemental Figure 4…………………………………………………………….S8

Supplemental Figure 5…………………………………………………………….S9

Supplemental File 1……………………………………………………………..S9

Supplemental File 2……………………………………………………………S10

Supplemental File 3……………………………………………………………S13

Supplemental File 4……………………………………………………………S16

Supplemental Table 1. Treatment characteristics for AFTER study patients (male, pre-menopausal and post-menopausal. Statistical tests carried out between pre- and post-menopausal patients.

Supplemental Table I. Treatment Characteristics

Variable	Male<br>N = 2<br>No. (%)	Premenopausal<br>N = 14<br>No. (%)	Postmenopausal<br>N = 12<br>No. (%)	*P**
Radiotherapy after surgery				0.101[b]
YES	0 (0.0)	4 (14.3)	8 (28.6)	
NO	2 (7.1)	10 (28.0)	4 (14.3)	
Chemotherapy after surgery				0.403[b]
YES	1 (3.6)	3 (10.7)	5 (17.9)	
NO	1 (3.6)	11 (39.3)	7 (25.0)	
Hormonal therapy after surgery				0.158[b]
YES	2 (7.1)	9 (32.1)	11 (39.3)	
NO	0 (0.0)	5 (17.9)	1 (3.6)	
Type of hormonal therapy after surgery				0.035[b]
anastrozole	0 (0.0)	0 (0.0)	3 (10.7)	
tamoxifen	2 (7.1)	9 (32.1)	5 (17.9)	
tam + anastrozole	0 (0.0)	0 (0.0)	2 (7.1)	
tam + exemestane	0 (0.0)	0 (0.0)	1 ((3.6)	
NA	0 (0.0)	5 (17.9)	1 (3.6)	
Type of surgery				0.238[b]
mastectomy	0 (0.0)	4 (14.3)	7 (25.0)	
wide-local excision	2 (7.1)	10 (28.0)	5 (17.9)	

*P**Tests are performed only on premenopausal and postmenopausal data; [a]Wilcoxon-rank-sum-test; [b]Pearson's chi-squared test

Supplemental Table 2. Additional characteristics of patient samples for which we obtained post-treatment ChIP-seq data.

Supplemental Table II. Characteristics of Post-treatment Paired Samples

Variable	Case 1	Case 2	Case 3	Case 4
Age	64	58	73	47
Tumor size (cm, ultrasound)	20	30	18	NA
Histological subtype	IDC	IDC	IDC	IDC
Nodal status therapy after surgery	negative	negative	positive	micrometastasis
Histological grade	2	1	3	3
ER percentage baseline	100	70	50	50
PR percentage baseline	100	70	50	50
Ki67 percentage baseline	10	20	20	20
Ki67 percentage after TAM	1	5	10	10
MKI67 gene expression direction after TAM	down	down	down	down
TAM exposure duration (days)	16	35	26	11

NA indicates no data; cm indicates centimeters; TAM indicates tamoxifen

Supplemental Figure 1

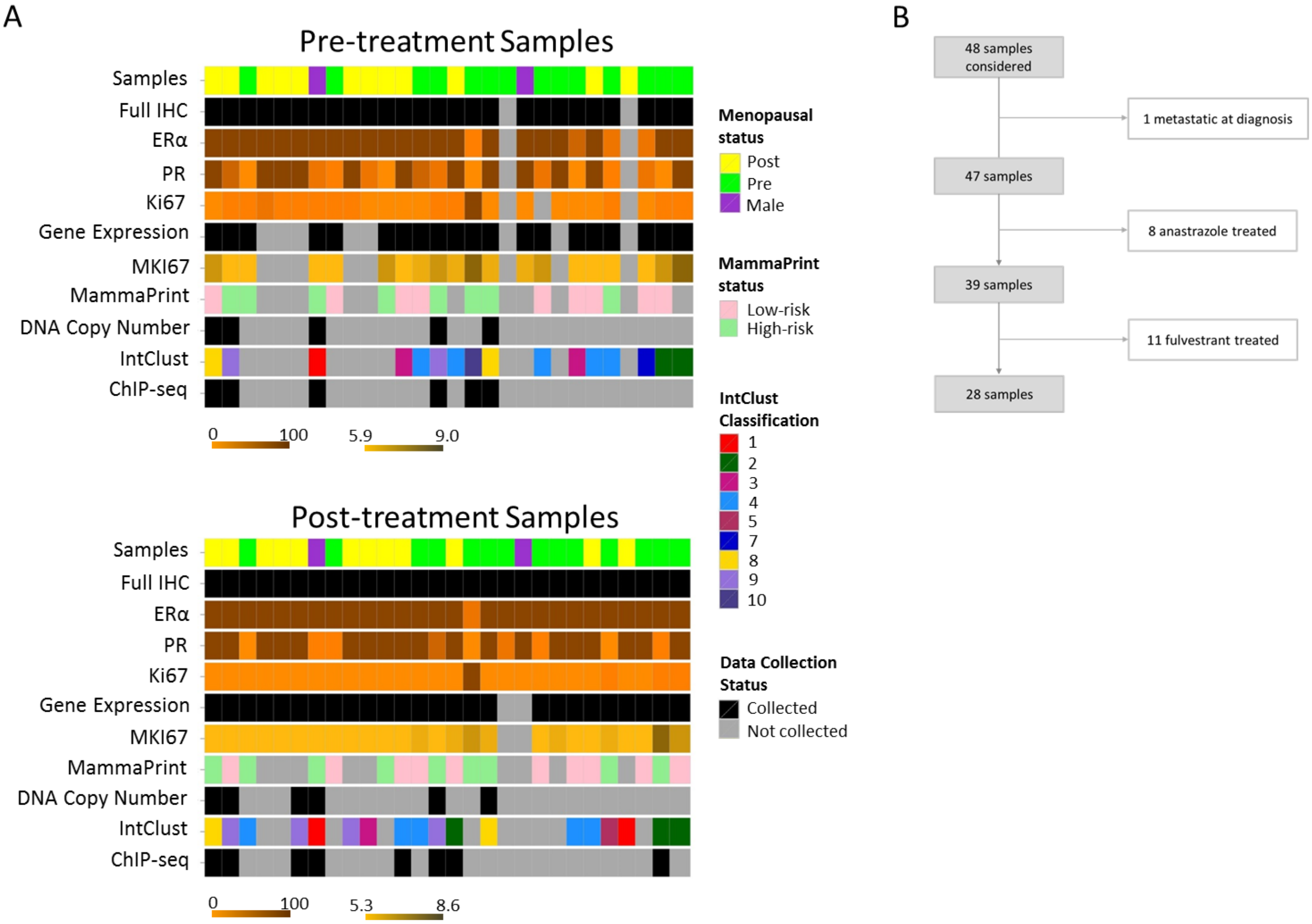

Supplemental Figure 1. Colormap of data collection. A, Colormap indicating data collected for all 28 individuals (top panel; pre-treatment, bottom panel; post-treatment). Each box represents one sample, with color indicating data information in the legend. Full IHC indicates samples with ERα, PR and Ki67 scores available. From light orange (0%) to dark orange (100%), the specific IHC percent nuclei scores for each are also shown. The gene expression values for MKI67 are shown from low (yellow) to high (dark yellow). B, A consort diagram depicting patient collection.

Supplemental Figure 2

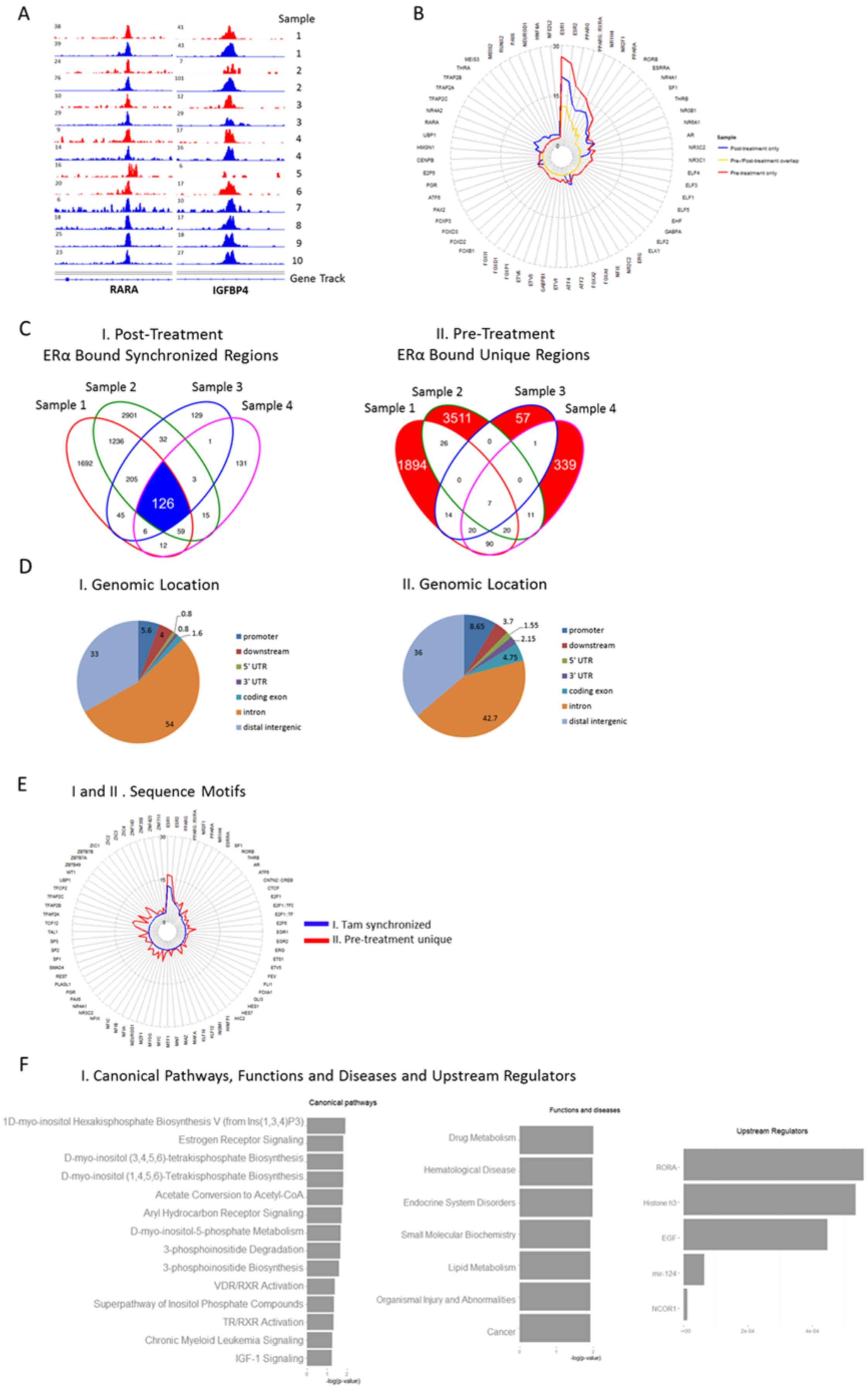

Supplemental Figure 2. Characteristics of ERα binding regions. A, Example of two ERα bound regions with read counts found in the human genome for all samples. Red and blue indicate pre- and post-treatment samples, respectively. B, Radial plot depicting comparative motif enrichment of the ERα binding regions for samples that are pre- (red), post-treatment (blue) and the overlapping regions (yellow). Enriched motifs (outside ring) and their z-score significance are shown (inside). For easier interpretation, z-score is used after taking absolute values. Original z-score threshold set at -3.09. C, Venn diagrams depicting overlapping peaks (paired samples) for both post-treatment tamoxifen synchronized sites (I) and unique pre-treatment sites (II). D, CEAS defined genomic regions enriched for sites I and II. E, Radial plot depicting comparative motif enrichment for sites I (red) and II (blue). Enriched motifs (outside ring) and their z-score significance are shown (inside). For easier interpretation, z-score is used after taking the absolute values, with original z-score threshold set at -3.09. F, Ingenuity Pathway Analysis of the 96 genes associated with the 126 tamoxifen synchronized regions. Barplots show most significantly associated Canonical Pathways, Diseases and Functions and Upstream Regulators.

Supplemental Figure 3

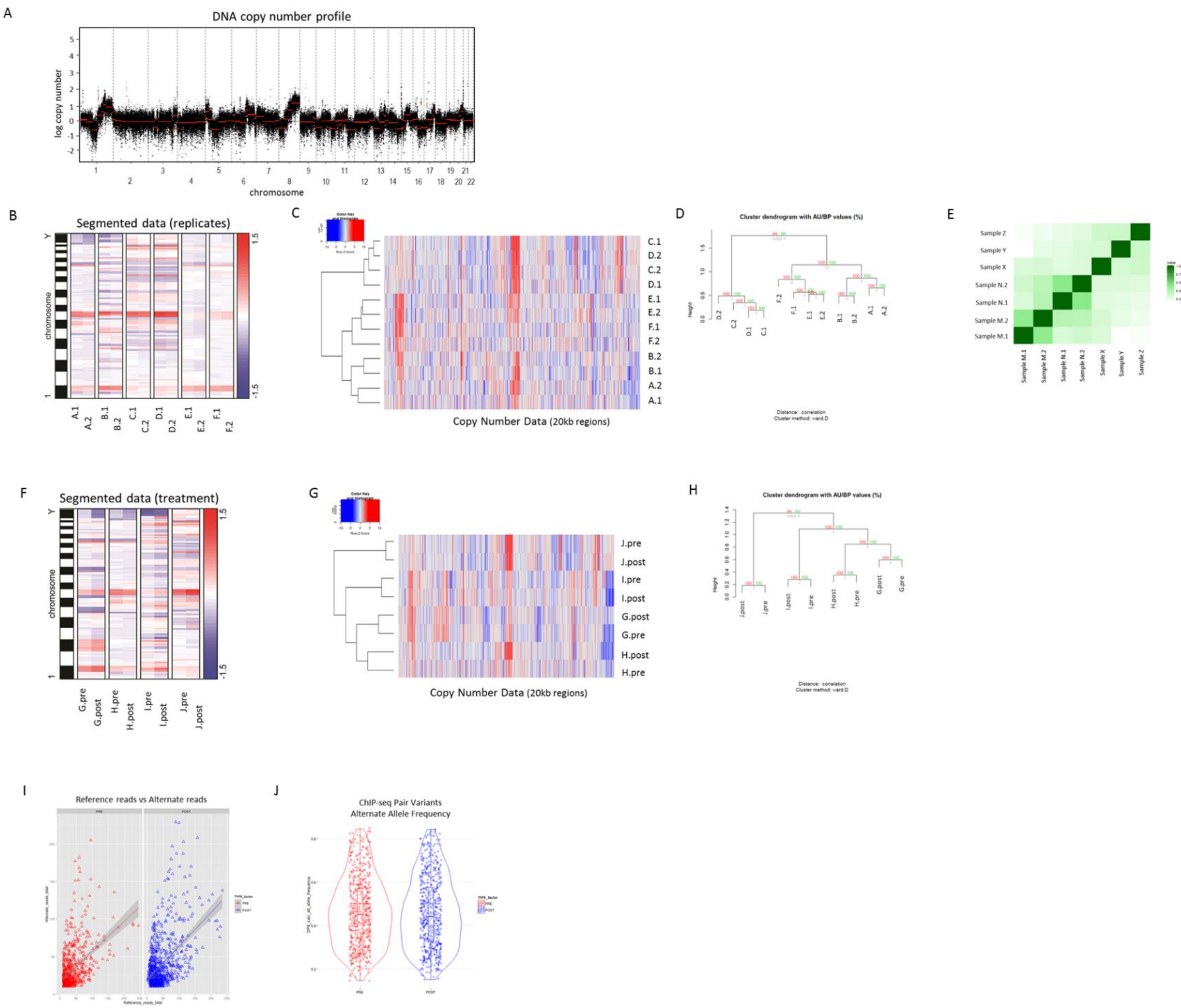

Supplemental Figure 3. DNA copy number cluster and alternate allele analysis. A, Example of a DNA copy number profile generated with the CopywriteR package. Chromosomes are on the X-axis (chromosomes X and Y not shown) and the log2 copy number is on the Y-axis.B, Segmentation values of the copy number data are represented as a heatmap showing the replicate samples. F, Pre- and post-treatment samples. Log2 ratio scale bar is depicted on the right of both graphs. Chromosomes shown on the left. C, Hierarchical clustering of DNA copy number data for sample replicates. Replicates are indicated by a suffix 1 or 2. Scaled expression value is denoted as the row z-score and plotted in red–blue color scale. Red indicates high expression and blue low expression. D, Dendrogram depiction of 'pvclust' results showing the relationship of replicate samples with AU *p*–values in red and BP *p*-values in green. G, Hierarchical clustering of DNA copy number data for pre- and post-treatment sample pairs. Treatment pairs are indicated by a suffix pre or post. Scaled copy number value is denoted as the row z-score and plotted in red–blue color scale. Red indicates copy number gain and blue copy number loss. H, Dendrogram depiction of 'pvclust' results showing the relationship of pre- and post-treatment samples with *p*-values shown as above. E, Correlation matrix (Spearman's *rho*) representing correlation of each combination of samples with respect to read count in 2,262 known non-overlapping ERα binding regions. Darker green indicates better correlation. Replicate pair samples are indicated with a suffix 1 or 2. I, Scatterplot indicating all alternate and reference allele counts (paired or non-paired) for both pre- (red) and post-treatment (blue) samples. J, Boxplot with violin plot overlay of DP4 calculated alternate allele frequency in paired variants for each of 4 paired samples. Pre-treatment sample variants are shown in red, post-treatment variants are shown in blue.

Supplemental Figure 4

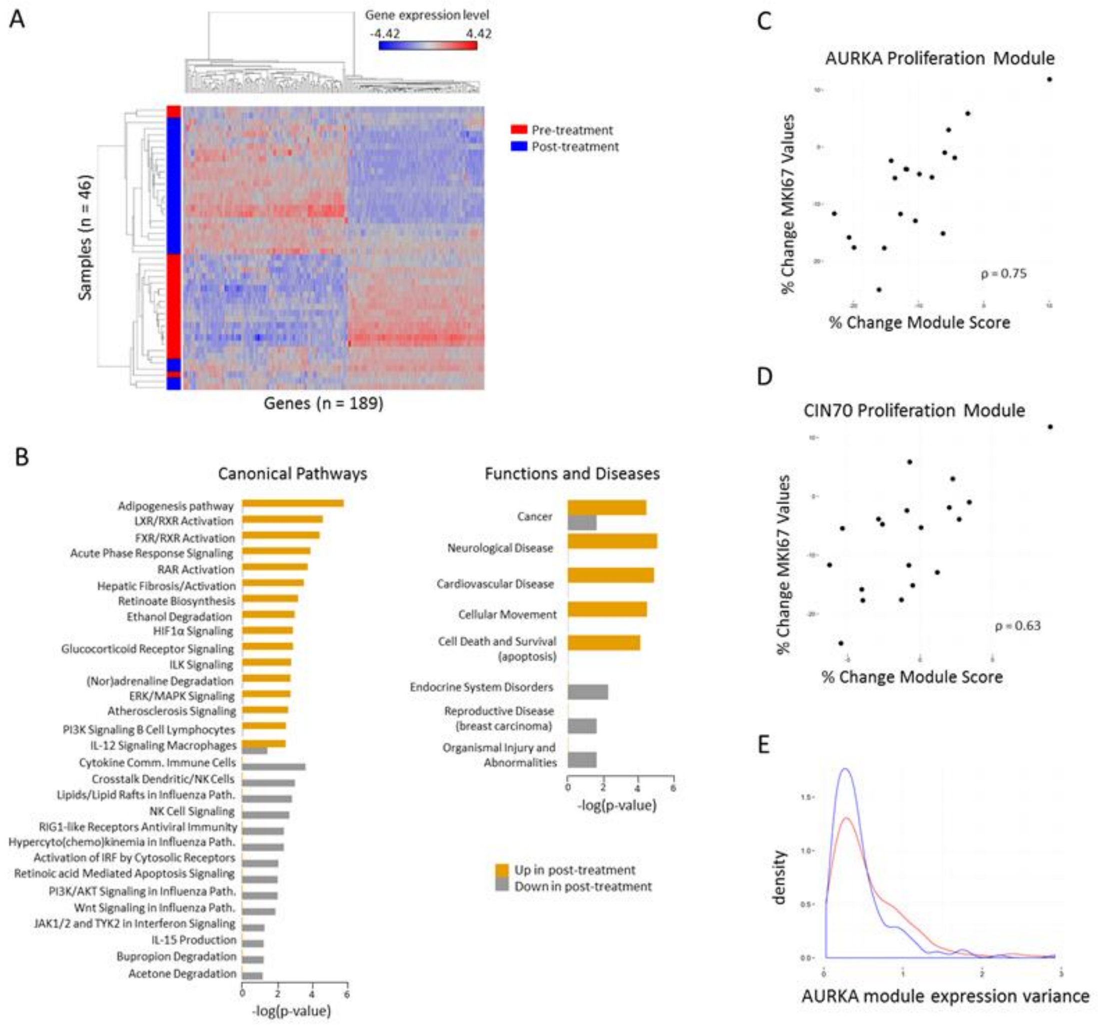

Supplemental Figure 4. Gene expression analysis. A, Hierarchical clustering of 189 top variable genes most differentially expressed between treatment condition (ANOVA, FDR<0.001, fold-change>2) in 46 breast tumors. Standardized gene expression level is depicted with high expression (red) and low expression (blue). Information column on the left depicts pre-treatment samples in red and post-treatment samples in blue. B, Ingenuity Pathway Analysis of the two groups of genes, upregulated in post-treatment (gold) and down-regulated in post-treatment (grey). Barplots show most significantly associated Canonical Pathways and Diseases and Functions. C, Scatterplot of cell proliferation module score (AURKA) percent change between pre- and post-treatment vs MKI67 gene expression percent change between pre- and post-treatment.D, Scatterplot of cell proliferation module score (CIN70) percent change between pre- and post-treatment vs MKI67 gene expression percent change between pre- and post-treatment. E, Density diagram depicting the gene expression variance for pre- and post-treatment conditions of the genes found in the AURKA cell proliferation module. Density plot of variance of pre-treatment (red) and post-treatment (blue) AURKA gene expression of signature module genes.

Supplemental Figure 5

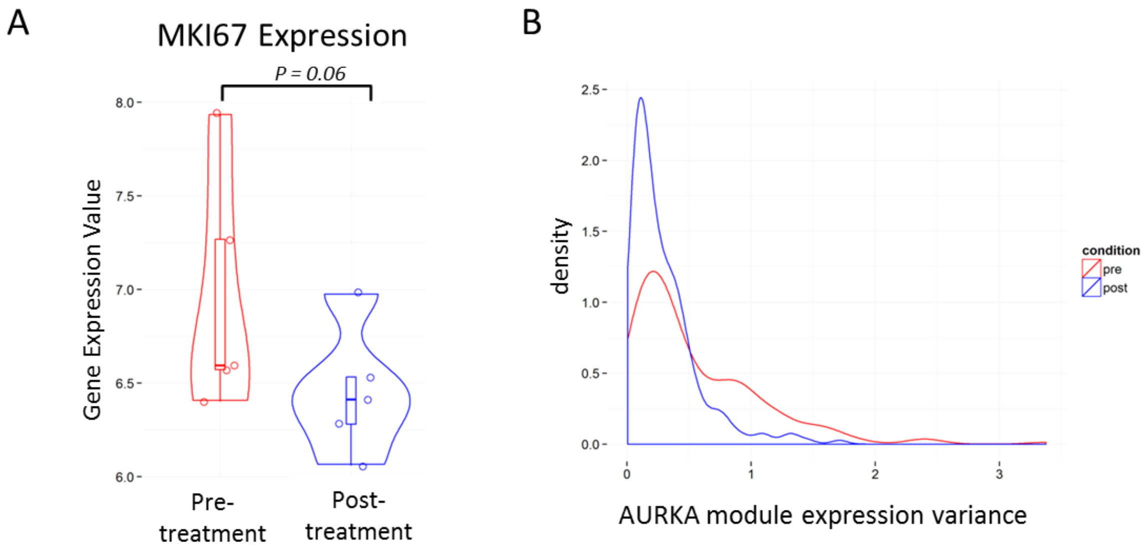

Supplemental Figure 5. Separate analysis of sample pairs with less than 2 weeks tamoxifen treatment. A, Violin plots with boxplot overlay of MKI67 gene expression values in pre- and post-treatment samples. B, Density diagram depicting the gene expression variance for pre- and post-treatment conditions of the genes found in the AURKA cell proliferation module. Density plot of variance of pre-treatment (red) and post-treatment (blue) AURKA gene expression of signature module genes.

Supplemental File 1. MammaPrint and IntClust classifications.

In breast cancer, the MammaPrint test can classify patients into two risk groups, low or high risk of metastasis (van ’t Veer et al., 2002). For 17 paired samples with MammaPrint results, most had the same classification between pre- and post-treatment samples (7 remained Low-Risk and 7 remained High-Risk). Three samples however, did change in result (2 samples from Low- to High-Risk during treatment and 1 sample changed from High- to Low-Risk.) (Supplemental Figure 1A). An additional classification method that uses both gene expression and DNA copy number data also classifies breast cancer samples into 10 different groups associated distinct clinical outcomes (Ali et al., 2014; Curtis et al., 2012). Using our gene expression and DNA copy number data we classified the samples into the 10 IntClust groups (Ali et al., 2014; Curtis et al., 2012). IntClust classifications were available for 13 paired samples. We found 9 of 13 pairs did not change class and 4 pairs did change class: (i) two from IntClust3 to IntClust4. IntClust3 is a class characterized by 68% Luminal A and 15% Luminal B samples. Inclust4 is a class with 31% Luminal A and 8% Luminal B samples. Both IntClust3 and IntClust4 classes are associated with good prognosis. (ii) One sample switched from IntClust4 to IntClust2, a class characterized by 35%

Luminal A and 50% Luminal B samples. The IntClust2 class is characterized by the worst prognosis among ERα-positive tumors. (iii) Finally, one sample changed from IntClust4 to IntClust5 a class which contains only 5% Luminal A and 17% Luminal B but 57% HER2-like samples. Prognosis for HER2-like samples was very poor before the use of anti-HER2 treatments (Ali et al., 2014; Curtis et al., 2012) (Supplemental Figure 1A). For all samples, both pre- and post-treatment samples were enriched for IntClust4 classification, typically representing ERα-positive, Luminal A samples ($P < 0.001$ and 0.01, respectively, Pearson's chi-square test) (Curtis et al., 2012).

Supplemental File 2. A bed file containing the post-tamoxifen treatment synchronized regions (hg19)

chr1	1008991	1009660
chr1	7507760	7508391
chr1	16934741	16935310
chr1	17846810	17847410
chr1	21568060	21568641
chr1	30934341	30935010
chr1	31841460	31842060
chr1	42255310	42255910
chr1	44533091	44533741
chr1	51786910	51787541
chr1	91565510	91566191
chr1	2.03E+08	2.03E+08
chr1	2.03E+08	2.03E+08
chr1	2.03E+08	2.03E+08
chr2	1680048	1680600
chr2	11638598	11639200
chr2	70203598	70204150
chr2	1E+08	1E+08
chr2	2.38E+08	2.38E+08
chr3	50164443	50165174
chr3	58452743	58453393
chr3	1.56E+08	1.56E+08
chr3	1.69E+08	1.69E+08
chr3	1.94E+08	1.94E+08
chr3	1.94E+08	1.94E+08
chr4	1239086	1239695
chr4	1827536	1828095
chr4	1.41E+08	1.41E+08
chr5	1.32E+08	1.32E+08
chr5	1.33E+08	1.33E+08

chr5	1.39E+08	1.39E+08
chr5	1.74E+08	1.74E+08
chr6	1.49E+08	1.49E+08
chr7	906636	907286
chr7	921936	922486
chr7	1976136	1976936
chr7	2579036	2579586
chr7	45915736	45916436
chr7	47581536	47582136
chr8	67434545	67435207
chr8	98776357	98777015
chr8	1.04E+08	1.04E+08
chr8	1.29E+08	1.29E+08
chr8	1.29E+08	1.29E+08
chr8	1.29E+08	1.29E+08
chr8	1.44E+08	1.44E+08
chr8	1.44E+08	1.44E+08
chr9	33163693	33164221
chr9	33221121	33221743
chr9	92270821	92271493
chr9	1.31E+08	1.31E+08
chr9	1.31E+08	1.31E+08
chr9	1.38E+08	1.38E+08
chr10	5701850	5702557
chr10	6195157	6195657
chr10	71198950	71199507
chr10	79631850	79632557
chr10	1.15E+08	1.15E+08
chr10	1.16E+08	1.16E+08
chr10	1.27E+08	1.27E+08
chr10	1.29E+08	1.29E+08
chr11	1389306	1389980
chr11	1507006	1507680
chr11	17427180	17427780
chr11	35285706	35286306
chr11	69706230	69706880
chr11	72497330	72497956
chr11	75044630	75045306
chr12	2904979	2905579
chr12	1.17E+08	1.17E+08
chr12	1.22E+08	1.22E+08
chr12	1.24E+08	1.24E+08
chr12	1.25E+08	1.25E+08
chr12	1.25E+08	1.25E+08
chr12	1.25E+08	1.25E+08
chr12	1.25E+08	1.25E+08

chr12	1.25E+08	1.25E+08
chr13	1.15E+08	1.15E+08
chr14	50459496	50460096
chr14	93483582	93484196
chr14	94856896	94857532
chr16	1428769	1429328
chr16	2794578	2795219
chr16	83980319	83980969
chr17	26824901	26825451
chr17	38336838	38337451
chr17	38478338	38479001
chr17	38604788	38605499
chr17	55174001	55174551
chr17	55977001	55977651
chr17	56716901	56717599
chr17	72754238	72754851
chr17	72756651	72757338
chr17	75282738	75283351
chr17	80061688	80062301
chr17	80571788	80572459
chr17	80846792	80847551
chr17	80850801	80851351
chr18	24325222	24325772
chr19	1166675	1167275
chr19	1170125	1170725
chr19	1181473	1182193
chr19	4970623	4971225
chr19	4976225	4976923
chr19	7684623	7685283
chr19	18557625	18558275
chr19	18806725	18807325
chr19	34120325	34121173
chr19	35808223	35808875
chr19	38812123	38812775
chr19	39204925	39205573
chr19	41426875	41427673
chr20	20421788	20422356
chr20	24992238	24992806
chr20	35215438	35216156
chr20	36773206	36773888
chr20	43394056	43394656
chr20	46786638	46787206
chr20	47188606	47189206
chr20	56629706	56630441
chr21	16959062	16959699
chr22	38177443	38177943

chr22	43787143	43787806
chr22	43795806	43796406
chr22	45326943	45327456
chr22	48320843	48321456

Supplemental File 3. Additional description of the sample sets used for determining the known ER binding 'universe'.

To generate a 'universe' of known ER binding peaks from our analyses, we called peaks in public data as described in ChIP-sequencing analysis in the methods section and used DiffBind to determine the overlap rates of peaks among the samples.

In total 52 ERα ChIP-seq samples from breast tumors and cell lines were available from Ross-Innes et al., Hurtado et al., Kraus et al., Lupien et al., papers (references in main text). Our own data were added to the dataset.

Number of peaks:

ID	Dataset	Factor	# Peaks
GSM798383	Ross-Innes	ERα	5950
GSM798384	Ross-Innes	ERα	4858
GSM798385	Ross-Innes	ERα	3795
GSM798386	Ross-Innes	ERα	24427
GSM798387	Ross-Innes	ERα	1218
GSM798388	Ross-Innes	ERα	847
GSM798389	Ross-Innes	ERα	3413
GSM798390	Ross-Innes	ERα	211
GSM798391	Ross-Innes	ERα	1145
GSM798392	Ross-Innes	ERα	1597
GSM798393	Ross-Innes	ERα	40843
GSM798394	Ross-Innes	ERα	2788
GSM798395	Ross-Innes	ERα	25271
GSM798396	Ross-Innes	ERα	1005

GSM798397	Ross-Innes	ERα	5098
GSM798398	Ross-Innes	ERα	3331
GSM798399	Ross-Innes	ERα	7798
GSM798400	Ross-Innes	ERα	1768
GSM798401	Ross-Innes	ERα	9566
GSM798402	Ross-Innes	ERα	65115
GSM798403	Ross-Innes	ERα	10464
GSM798423	Ross-Innes	ERα	57955
GSM798424	Ross-Innes	ERα	39704
GSM798425	Ross-Innes	ERα	50199
GSM798426	Ross-Innes	ERα	48647
GSM798427	Ross-Innes	ERα	28743
GSM798428	Ross-Innes	ERα	16428
GSM798429	Ross-Innes	ERα	6681
AFTER001_pre	Severson	ERα	2071
AFTER002_pre	Severson	ERα	3575
AFTER013_pre	Severson	ERα	100
AFTER022_pre	Severson	ERα	490
AFTER026_pre	Severson	ERα	8240
AFTER027_pre	Severson	ERα	536
AFTER001_post	Severson	ERα	3381
AFTER002_post	Severson	ERα	4577
AFTER010_post	Severson	ERα	273
AFTER013_post	Severson	ERα	574
AFTER020_post	Severson	ERα	170
AFTER022_post	Severson	ERα	358
AFTER024_post	Severson	ERα	475

AFTER050_post	Severson	ERα	8108
GSM1534720	Hurtado	ERα	1038
GSM1534721	Hurtado	ERα	586
GSM1534722	Hurtado	ERα	16437
GSM1534723	Hurtado	ERα	18887
ERR022025	Kraus	ERα	2320
ERR022052	Kraus	ERα	7850
ERR022053	Kraus	ERα	7235
ERR022056	Kraus	ERα	977
ERR022057	Kraus	ERα	3575
Lupien	Lupien	ERα	10577

Number of peaks as a function of the number of samples they appear:

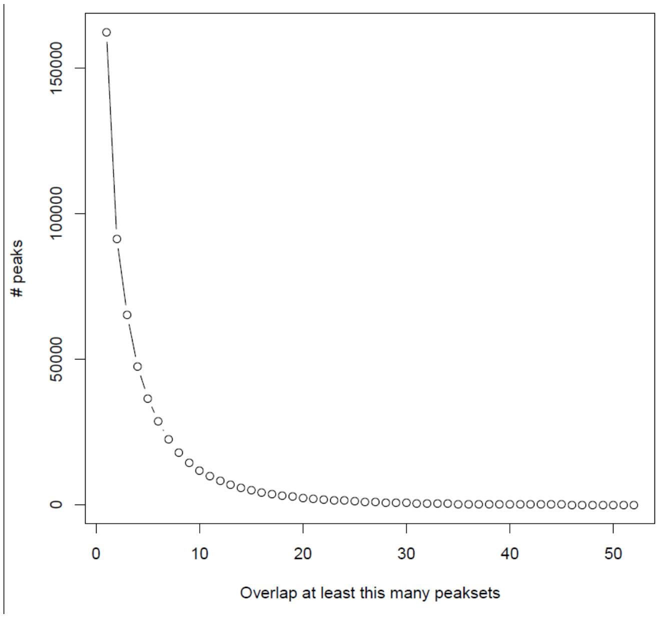

A consensus list was created:

Peaks present in at least one sample N = 162,449

Supplemental File 4. File containing all the differentially expressed genes (pre-treatment vs post-treatment with FDR < 0.05) with log2 fold change and ANOVA p-value

Gene/probe	ANOVA p-value	log2 Fold change
DUSP1	2.18E-11	9.9683
CYR61	9.83E-11	3.97855
ZFP36	2.55E-10	3.79048
NR4A1	8.66E-10	10.8189
ATF3	1.88E-09	5.18188
EGR1	1.88E-09	7.16391
FOS	7.63E-09	10.9931
ENST00000469435	2.51E-08	3.8355
SPRY1	3.82E-08	3.07056
C10orf10	1.22E-07	5.24707
RGS2	1.61E-07	3.01911
UNQ9368	2.48E-07	4.00276
ALDH1A1	2.48E-07	3.11674
EGR2	2.57E-07	3.51837
APOLD1	2.86E-07	3.19316
KLF6	3.30E-07	2.94167
ADH1A	5.17E-07	4.1892
TMEM49	7.21E-07	2.97837
RGS1	8.65E-07	3.69291
W60781	9.36E-07	8.09527
JUN	9.36E-07	3.60015
SLC2A3	9.62E-07	2.97125
CRYAB	1.16E-06	4.30838
FGF2	1.78E-06	3.3856
GOLGA6A	2.07E-06	-2.79546
OR2T8	2.17E-06	-2.72258
ADM	2.17E-06	3.56718
SLC6A9	2.56E-06	4.98748
DST	2.58E-06	3.30202
AKR1C3	2.65E-06	2.83425
LPL	2.68E-06	4.48377
SERPINE1	2.68E-06	4.3892
MUC17	2.82E-06	-3.81355
CDC27	2.82E-06	-5.02753
GSN	3.17E-06	2.76543
FHL1	3.23E-06	4.24668
GPX3	3.44E-06	2.7195
CFD	4.17E-06	3.24279
ITIH5	5.30E-06	2.92284
LOC387763	5.37E-06	3.11074
CHRDL1	5.50E-06	2.8845

OR5H14	5.83E-06	-3.36043
PRG1	5.83E-06	2.92453
LOC729706	6.49E-06	-3.51499
CLEC3B	7.14E-06	3.43735
ENST00000440673	7.14E-06	-3.31268
SAA2	7.26E-06	5.63649
SOD3	7.26E-06	2.65931
LOC100134409	7.26E-06	-4.92736
APOD	7.54E-06	4.43407
KIR2DS2	7.88E-06	-3.46126
SAA1	7.90E-06	6.89126
KIR2DL4	8.53E-06	-2.54024
DHRS8	9.12E-06	2.53243
P11	9.12E-06	-2.75857
REXO1L1	9.91E-06	-5.66942
OTOP1	1.14E-05	-2.88356
MUC12	1.16E-05	-3.34184
KIR2DS1	1.28E-05	-2.66014
FKSG73	1.29E-05	-2.76525
SRGN	1.37E-05	2.7418
ADH1C	1.64E-05	3.26462
IRX1	1.65E-05	2.66558
CD36	1.68E-05	3.14106
DRD5	1.79E-05	-3.10529
W95609	1.79E-05	2.58864
NDRG2	2.02E-05	2.65887
TRIM64	2.09E-05	-2.65881
ANGPTL4	2.35E-05	2.81667
ZFP36L2	2.57E-05	2.86361
PRAMEF4	2.58E-05	-5.17625
FAM75A3	2.59E-05	-2.76456
U88898	2.73E-05	-3.83946
A_23_P21045	2.84E-05	-2.63235
KIR2DL2	3.04E-05	-2.93925
UBTFL1	3.04E-05	-2.52213
LOC647322	3.04E-05	-2.78234
FOXD4L2	3.14E-05	-2.98364
NRP1	3.37E-05	2.54924
IFNA6	3.37E-05	-2.92013
USP17	3.73E-05	-4.059
ERRFI1	3.74E-05	2.49781
A_23_P384418	3.74E-05	-3.4946
LOC339524	3.81E-05	2.59991
PLIN2	3.81E-05	2.41831
PI16	3.90E-05	5.25723
SORBS1	4.17E-05	2.82867

C1orf186	4.22E-05	-2.7238
EBF1	4.23E-05	2.43334
GOLGA6L6	4.51E-05	-2.70944
tcag7.1213	4.56E-05	-2.46323
CYP1A2	4.56E-05	-2.68955
PRAMEF15	4.75E-05	-4.25563
SSX4B	4.76E-05	-2.87576
PPARG	5.26E-05	2.71103
PRAMEF5	5.39E-05	-3.32519
IFNA5	6.07E-05	-2.84164
ENST00000391545	6.07E-05	-3.28322
TNS1	6.23E-05	2.43912
F3	6.24E-05	2.97188
LOC645146	6.42E-05	-3.8211
TRIM53	6.42E-05	-3.61621
TIMP3	6.93E-05	3.18008
LOC652859	7.27E-05	-3.41235
HDGFL1	7.67E-05	-2.33142
DEFA1	8.02E-05	-3.16871
ADAM6	8.22E-05	-2.81046
U88897	8.22E-05	-2.52474
SERPINB10	8.30E-05	-2.91283
GGT8P	8.35E-05	-2.49262
CPM	8.45E-05	2.33728
SSX2	8.97E-05	-2.32279
DLX1	9.04E-05	-2.45739
C2orf40	9.06E-05	2.71728
FLJ36000	9.11E-05	-2.34765
LOC100133402	9.27E-05	-2.6407
LOC399900	0.000100083	-2.73504
HIF1A	0.00010413	2.48137
CFHR1	0.000104767	-2.3253
CIDEC	0.000105086	2.69824
SPRYD5	0.00011848	-2.93858
BTG2	0.000119725	2.40221
NM_013315	0.000119854	-2.98567
PRAMEF13	0.000132516	-2.94834
TGFBR3	0.00014215	3.23922
G0S2	0.000143466	4.41569
OR7E91P	0.000151521	-2.34442
ANXA1	0.00015877	2.51059
CCL3	0.00015877	2.56877
NHEDC1	0.000162106	-3.27243
DARC	0.000163464	2.5172
ENST00000400767	0.000163464	-2.3218
POU2AF1	0.000163464	2.80619

DUSP6	0.000188898	2.23914
KCNIP2	0.000188898	2.36432
PLIN4	0.000189693	2.32056
SSX1	0.000193658	-2.45694
CSAG3	0.000208439	-2.25545
IL20	0.000223332	-2.40644
TMPRSS11B	0.000235909	-2.44279
ZNF705G	0.000235909	-3.09627
A_32_P785717	0.000243398	-2.24497
ENST00000433626	0.00025684	-2.64941
MYH11	0.000284744	2.26233
HSPB7	0.000287959	2.19809
SRPX	0.000307167	2.19084
VIM	0.000352358	2.18654
LOC100133408	0.000353829	-2.30305
A_24_P917886	0.000360801	-2.66329
S1PR1	0.000414086	2.31572
NP100332	0.000427187	2.52096
GAGE7	0.000427187	-2.25745
RAB1A	0.000432328	2.27252
EFEMP1	0.000432328	2.3549
KRTAP21.1	0.000452753	-2.2116
RIMS2	0.000452753	-2.37029
CLDN11	0.000468354	2.72013
AQP7P1	0.000478802	2.52512
AI247465	0.000489005	2.35268
USP17L2	0.000489005	-2.36728
MGC39584	0.000525628	-2.37439
TMEM37	0.000534533	2.19078
A_24_P887857	0.000539871	2.33712
PSG2	0.000539871	-2.31712
KRT5	0.000540482	3.01373
KLF2	0.000540482	2.31471
HLF	0.000540672	2.35941
EDN1	0.00057116	2.29086
DGAT2	0.000579115	2.91934
FCAMR	0.000588875	-2.16379
EGR3	0.000590653	2.55066
LOC645032	0.000590653	-2.24716
KRTAP4.11	0.000597784	-2.19212
MFAP4	0.000604303	2.70227
TRIM49	0.000678193	-2.38207
H2BFM	0.000696832	-2.25456
COL17A1	0.000715481	3.51463
SLC39A14	0.00073782	2.14089
SH3KBP1	0.000765425	2.1045

OR5AK2	0.000785958	-2.39092
KRTAP19.1	0.000809407	-2.45281
ANTXR2	0.00085639	2.13679
MUC4	0.000859186	-2.3437
ENST00000441574	0.000874437	-2.33139
TF	0.000883808	2.26688
LOC642707	0.000898324	-3.39764
PTPRC	0.000933187	2.15297
LOC100133732	0.000941474	-2.21658
A_24_P239049	0.000987854	-2.27384
